# Pharmacokinetic Parameters and Over-Responsiveness of Iranian Population to Propranolol

**DOI:** 10.15171/apb.2017.024

**Published:** 2017-06-30

**Authors:** Ebrahim Salehifar, Shima Ebrahim, Mohammad-Reza Shiran, Fatemeh Faramarzi, Hossein Askari Rad, Razieh Avan, Asadollah Mohseni Kiasari, Pouneh Ebrahimi

**Affiliations:** ^1^Pharmaceutical Research Center, Faculty of Pharmacy, Mazandaran University of Medical Sciences, Sari, Iran.; ^2^Student Research Committee, Faculty of Pharmacy, Mazandaran University of Medical Sciences, Sari, Iran.; ^3^Immunogenetics Research Center, Faculty of Medicine, Mazandaran University of Medical Sciences, Sari, Iran.; ^4^Student Research Committee, Department of Clinical Pharmacy, Mazandaran University of Medical Sciences, Sari, Iran.; ^5^Faculty of Pharmacy, Mazandaran University of Medical Sciences, Sari, Iran.; ^6^Faculty of Medicine, Mazandaran University of Medical Sciences, Sari, Iran.; ^7^Department of Chemistry, Faculty of Basic Sciences, Golestan University, Gorgan, Iran.

**Keywords:** Propranolol, Pharmacokinetics, Pharmacodynamic, Iranian Population, Polymorphism

## Abstract

***Purpose:*** Propranolol is the most widely used treatment for cardiovascular diseases. Dosage range in our patients is usually less than the amount mentioned in references. The aim of the present study was to clarify whether pharmacokinetic differences are able to justify the need for the fewer doses in our patients or not.

***Methods:*** Twenty healthy volunteers (10 male) at heart center of Mazandaran University of Medical Sciences were studied. Samples of blood were collected before a single oral dose (40 mg) of Propranolol. Blood samples were taken up to 9 hours after dose. Total plasma concentration of Propranolol was measured by HPLC. Population Pharmacokinetic analysis was performed using population pharmacokinetics modeling software P-Pharm.

***Results:*** The mean value for oral plasma clearance (CL/F) was 126.59 ml/hr. The corresponding values for apparent volume of distribution (V/F), t1/2 beta, maximum blood concentration (C max), and time to reach the maximum blood concentration (T max) were 334.12 Lit, 1.98 hr, 40.25 ng/ml, and 1.68 hr, respectively. The observed mean values of V/F of propranolol in the present study were comparable with those reported in the literature. However, the mean values of CL/F of propranolol in current study was significantly higher than those reported in other population (P-value<0.001).

***Conclusion:*** This study has confirmed that the pharmacokinetic differences are not able to justify over-responsiveness of Iranian population to propranolol. Pharmacodynamic differences in responding to beta blocker drugs by Renin secretion or having a different sensibility to beta receptors might play a role in making our population have a different response to propranolol.

## Introduction

Propranolol is a nonspecific beta-blocker that was presented as the first beta blocker in 1960.^1^ It is used in various cases, and mainly to treat high blood pressure,^2^ cardiac arrhythmias,^3,4^ thyrotoxicosis,^5^ migraine headaches,^6^ and psychiatric diseases.^7^ In recent studies, the beneficial effects of the drug to inhibit angiogenesis,^8^ treatment of different types of cancer,^9-12^ and hemangioma in children^13^ has been established. The dosage range of propranolol is very broad and the maintenance dose and maximum dose listed in reference books are much higher than the usual dose in our patients. For example, the recommended dose of propranolol at the beginning of the treatment of hypertension is mentioned to be 80 milligrams per day and the gradual increase to the maintenance dose of 80-320 mg per day.^14^ Many complications such as hypotension, bradycardia, depression, fatigue, sexual disorders and weight gain have been reported after taking beta blockers so the drug at the proper dose is necessary.^15,16^ Despite the widespread use of propranolol, the suitable dosage range is not clear in Iranian population. It is believed that our patients are not able to tolerate high dosage of propranolol that is mentioned in the references. It seems that the intensification of pharmacodynamic effects of the drug, especially bradycardia or hypotension prevents prescribing a standard dose of the medication. The mean pharmacokinetic parameters of propranolol in healthy volunteers in previous studies, (clearance equal to 0.96‏ ±0.3 Lit/kg/hr, the volume of distribution equal to 4.3‏ ±0.6 Lit/kg, half-life equal to 3.4 ±0.4 (hour) and oral bioavailability equal to 26±10%), has been declared.^17,18^ Given the uncertainty of pharmacokinetic parameters of propranolol in our population, this study was conducted to determine pharmacokinetic parameters of propranolol in a sample of healthy volunteers of Iranian population.

## Materials and Methods

This study was conducted on 20 healthy volunteers (10 men) at the Fatemeh Zahra educational hospital of Mazandaran University of Medical Sciences. Each subject gave his or her written informed consent to participation in the study, which was approved by the Research Ethics Committee of Mazandaran University of Medical Sciences (approval number 2.3.84-458).

Those who agreed to participate underwent a short clinical examination and gave details of their age, sex, weight, and their medical history was collected. Electrocardiography was performed on all the participants. Only healthy adults, non-smokers and non-consumers of any inhibitor drugs (e.g., cimetidine, ketoconazole, erythromycin, clarithromycin) or metabolism inducer (e.g., rifampin, phenytoin, carbamazepine and clarithromycin) in the past two weeks were recruited. Exclusion criteria included: pregnancy, bradycardia (heart rate less than 55 beats per minute), low blood pressure (systolic pressure below 110 mmHg or mean arterial pressure below 70 mmHg) and disapproval of health of various organs in physical examination. After an overnight fasting, a sample of 5 ml blood (analyzed to confirm abstinence) was collected. The subjects received a single oral dose of 40 mg tablet of propranolol (Tolid daroo) with 250 ml water. They were fasted over 2 h post-dose and peripheral venous blood samples (5 ml) were taken at 0.5, 1, 2, 3, 6, 8, and 9 hours post dose. After centrifugation for 5-min (1000 g), the plasma samples were stored at -20 °C until analysis. Urinary pH of the volunteers was measured at the 0 and 4 hours. The plasma concentration of propranolol was treated according to the method of Hermansson J et al.^19^ 500 ml of thawed sample was mixed with 250 µl of zinc sulfate (0.07 molar) and 250 µl of sodium Hydroxide (1 molar). The mixture was vortex-mixed for 2 min. After centrifugation for 15 min at 3000 rpm the upper layer was transferred to a 1 ml tube and centrifuged for the second time at 1100 rpm for 5 minutes to obtain a clear solution and a 100 µl aliquot for 3 consecutive times, injected onto the HPLC. The chromatographic separation of propranolol was performed on a C8 analytical column (5 μm particle size, L × I.D. 15 cm × 4.6 mm) using an isocratic mobile phase of water- acetonitrile-methanol (65:25:5, v/v), 0.3% triethylamine. The pH of mobile phase was adjusted to 3.5 by means of phosphoric acid 85% and degassed by Knaver degaser, and delivered at a flow-rate of 0.5ml /min. The detector used was a UV, set at 233 nm wavelength.

### Pharmacokinetics analysis

Pharmacokinetics analysis was carried out using population pharmacokinetics modeling software P-Pharm (P-Pharm., version 1.5. InnaPhase, Ceretil, France). Various population pharmacokinetic models were fitted to the data. Selection of the best model was based on the lowest value of the Akaike Information Criteria (AIC), visual inspection of residuals for systematic error and the predicted versus actual concentration plots. Initial estimates of the pharmacokinetics parameters were derived from values reported in the literature. Non parametric methods were used to calculate AUC and drug elimination constant (K_e_). Statistical analysis was performed using SPSS for Windows (ver.16, SPSS Inc., Chicago, USA). For comparing non-paired clinical data, an independent samples t-test was used. In all cases p < 0.05 was taken as statistically significant. 

## Results and Discussion

The demographic characteristics of volunteers in present study are summarized in Table 1. There were no significant differences in the characteristics of patients between males and females (independent sample t-test), except for weight, and height (p =≥0.046). The weight and height were significantly higher in males than in females (p<0.001).

**Table 1:** Characteristics of the participants who completed the study.

-	**Male** (N=10)	**Female** (N=10)	**P-value**
**Age (year)**	27.3 ± 5.8 ‏	27.5 ± 7.2‏	0.95
**Height (cm)**	174.9 ± 4.5 ‏	161 ± 3.9‏	<0.001‏
**Weight (kg)**	73.4 ± 5‏	59.9 ± 7.6‏	<0.001‏
BMI*	24 ±‏ 1.1‏	23.2 ± 3.3‏	0.46

*BMI: Body Mass Index = Weight _(kg)_ / [Height _(m)_] ^2^

**Figure 1:**
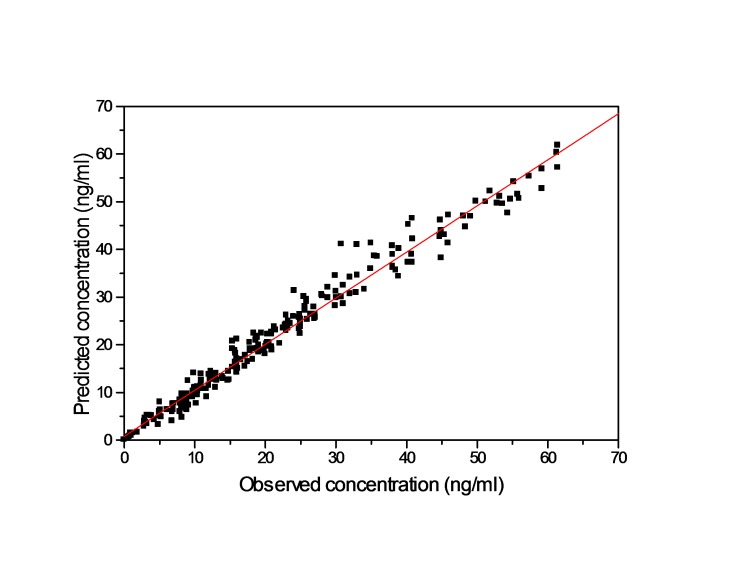
Population–derived Bayesian predicted vs observed total plasma concentrations after fitting of plasma concentration of propranolol calculated from the best fitted model

**Figure 2:**
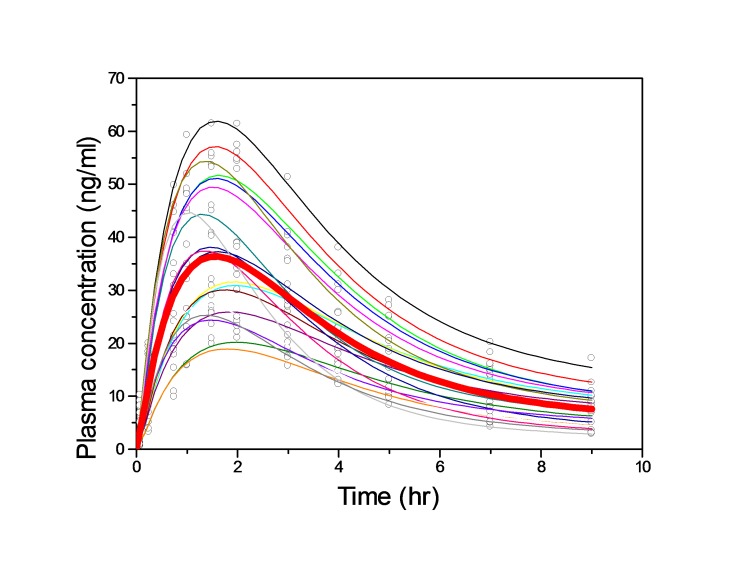
Population (bold line) and individual bayesian model fit to propranolol concentrations

A two-compartment pharmacokinetic model with first-order input, first-order distributional rate constants and first-order elimination provided a significantly better fit to the concentration-time profiles compared than other models. A heteroscedastic error model (1/y^2) was more appropriate for all the analytes. A lognormal distribution best described the inter-subject variability in all population pharmacokinetic parameters. The population–derived Bayesian predicted vs observed total plasma concentrations and population mean and individual bayesian model fit to propranolol concentrations are shown in Figure 1, and Figure 2, respectively.

Mean disposition and absorption pharmacokinetic parameter values for propranolol obtained from the best PK model and nonparametric analysis and the pharmacodynamics parameters are listed in Table 2 ‎, and Table 3, respectively.

With the exception of the Ka, there were no significant differences in any PK parameters of propranolol between males and females (p≤0.1). The Ka was significantly higher in females than in males (p=0.009).

Mean pharmacodynamic parameters including heart rate and blood pressure of the volunteers are listed in Table 3.

**Table 2:** Dose, pharmacokinetic parameters and urine pH of the participated volunteers

**Variable**	**Male (N=10)**			**Female (N=10)**			**P-value**
	**Mean±SD**	**Max**	**Min**	**Mean±SD**	**Max**	**Min**	
**‍Dose (mg/kg)**	0.55 ± 0.04	0.61	0.49	0.68 ±0.09	0.8	0.58	0.001
**Cp**_max_ ** (ng/ml)**	35.9 ± 12.1	56	22.1	44.6 ±14.5	61.4	21	0.17
**T**_max_ ** (min)**	105 ±16.6	120	90	96 ± 24	120	60	0.33
**Vd (Lit)**	360.9 ±114.8	609.4	221.7	307.4 ± 144.9	633.7	198.9	0.37
**Vd (Lit/Kg)**	4.95 ± 1.6	8.24	2.95	5.28 ±2.84	11.74	2.88	0.76
**Cl (Lit/hr)**	128.4 ±51.6	232.1	80	124.7 ±47.7	210.3	65.6	0.87
**Cl (Lit /Kg/hr)**	1.77 ± 0.79	3.36	1.03	2.17 ±1.02	4.12	0.95	0.34
**Ka**_(hr_^-1^_)_	0.5 ± 0.03	0.56	0.45	0.56 ± 0.06	0.66	0.47	0.009
**T½α (hr)**	0.77 ± 0.13	0.95	0.58	0.70 ± 0.14	0.9	0.44	0.28
**T½β (hr)**	19.7 ± 5.7	32.5	13.3	17.4 ± 6.4	33	12.21	0.4
**T½ (hr)**	2.1 ± 0.84	3.6	1.1	1.83 ± 0.75	3.1	0.67	0.41
**AUC**_0-2_ ** (ng×hr/ml)**	32.7 ± 11.4	51.8	16.3	43.8 ± 16.4	63.51	16.14	0.1
**AUC 0-10 (ng×hr/ml)**	169.2 ± 52.8	257.1	107.4	194.5 ±72.1	313.9	97.2	0.38
**AUC**_0-∞_ **(ng×hr/ml)**	169.6 ± 52.8	257.4	107.9	194.8 ± 72	314.1	97.8	0.38
**pH at time zero urine**	5.9± 0.74	7	5	5.7 ±0.82	7	5	0.57
**pH at time 4hr urine**	6.4 ± 0.52	7	6	6.1 ± 0.57	7	5	0.23

AUC: area under the curve; C_P_: concentration; CL: clearance; Vd: volume of distribution

**Table 3:** Heart rate and blood pressure of the volunteers after taking a single dose of 40 mg propranolol

**Variable (time; min)**	**Male (N=10)**			**P-value** ^€^ **(within group)**	**Female (N=10)**			**P-value** ^€^ **(within group)**	**P-value** ^¥^ ** (Between two groups)**
	**Mean±SD**	**Max**	**Min**		**Mean±SD**	**Max**	**Min**		
**SBP (0)**	115±13.5	130	90	P = 0.024 (for SBP)	111 ±16.6	130	90	0.56	P < 0.001 (for SBP)
**SBP (180)**	112±12.3	130	90		107 ± 14.2	120	90	0.41	
**SBP (360)**	112 ± 6.3	120	100		105±15.01	130	90	0.19	
**SBP (540)**	104 ± 8.4	110	90		99 ± 13.7	120	80	0.34	
**DBP (0)**	82 ± 9.2	90	60	P = 0.27 (for DBP)	79 ± 9.95	90	60	0.49	P = 0.55 (for DBP )
**DBP (180)**	82 ± 9.2	90	60		79 ± 9.95	90	60	0.49	
**DBP (360)**	84 ± 11.7	100	60		81 ± 7.4	90	70	0.5	
**DBP (540)**	80 ± 8.2	90	60		80 ± 9.4	90	60	1	
**HR (0)**	65.4 ± 1.9	68	62	P = 0.059 (for HR)	63.7 ± 2.9	67	60	0.15	P = 0.51 (for HR)
**HR (45)**	64.6 ± 2.6	68	60		63.2 ± 1.8	66	60	0.19	
**HR (180)**	64.3 ± 3.3	68	60		63.4 ± 2.5	68	60	0.56	
**HR (540)**	63.5 ± 1.4	65	62		62 ± 2.3	66	60	0.14	

DBP: diastolic blood pressure; HR: heart rate; SBP: systolic blood pressure; €: P-value of repeated measure test within each sex; ¥: P-value of independent samples t-test

Changes in systolic blood pressure, following the administration of propranolol in the measured times in both genders, was significant and in changes of heart rate in male volunteers, there was marginally significant (the trend toward significance) (P=0.059). In hemodynamic variables and their changes in different times, there was no significant difference between male and female volunteers. In Table 4 pharmacokinetic parameters resulted from our study was compared with the average reported in the references.^17,18^

**Table 4:** Comparison of pharmacokinetic parameters resulted from the current study with the average reported in the references

-	**This study**	**Other sources**	**Mean of differences**	**95% confidence interval**	**P-Value**
**Cl (Lit/kg/hr)**	1.97± 0.91	0.096±0.3	+1.01	0.59 to 1.44	<0.001
**Vd (Lit/kg)**	5.11± 2.3	4.3± 0.6	+0.82	0.24 to 1.87‏-	0.123
**t**_1/2_ ** (hr)**	1.98± 0.8	3.4± 0.4	-1.41	-1.79 to -1.05	<0.001

CL: clearance; Vd: volume of distribution

The obtained clearance in our study is more than the average reported in the references and half-life is less than the average reported in the references (P<0.001). The volume of distribution was not significantly different with the amounts reported in the references.

Based on the above mentioned results, propranolol clearance was more than the average reported in the references, while its half-life was less and there was no significant difference in the volume of distribution of the drug. Propranolol is metabolized to 14 metabolites, through 3 major pathways including Glucuronidation, side chain oxidation and ring-oxidation.^20^ Regarding the previous studies, cytochrome P450 2D6, is the main enzyme responsible for propranolol, that 50 to 90 percent of -4 hydroxylation of the drug is performed by this enzyme. Previous studies have shown that oral clearance of propranolol is higher in black people due to the higher activity of hepatic metabolic pathways.^21,22^ However in the study about the effects of 2D6 genotypes on the side effects and effectiveness of Metoprolol in the treatment of hypertension,^9^ despite the influence of these factors on pharmacokinetic of the drug, it was not associated with side effects of beta blocker or its effectiveness.^23^ In another study on the racial differences in pharmacokinetic propranolol, it was found that CYP1A2 metabolism pathway plays a role in 4-hydroxylation of propranolol, as well as CYP2D6, and the formation of metabolites resulting from these metabolic pathways, is considerably higher in African- American race than in Asian race. These racial differences in drug metabolism are related to the effectiveness or toxicity of the drug^24^ and pharmacokinetic parameters of propranolol. Although in our study, the minimum concentration (Cp_max_) in women was %24.2 more than men (44.6ng/ml against 35.1ng/ml). But these differences were not statistically significant. In the study of Xie and Chen in china, Cp_max_ in women and their AUC were respectively %99 and %74 more than men, the results were statistically significant. Moreover, half-life of the drug in women was longer than in men.^25^ In another study on the difference of propranolol metabolic clearance in both genders it has been observed that after administration of 80 mg of the oral drug, there has been no significant difference in volume of distribution and half-life of the drug. Nevertheless, plasma level of the drug after oral administration was higher in women than in men. These results correspond to the results from our study. In addition, Walle et al found that oral clearance of propranolol in women was considerably less than in men.^26^ In our study, the average half-life of Propranolol in women was not statistically different with men, though numerically half-life was lower in women (1.83 hr against 2.1 hr). Considering the equation t1/2=0.693*Vd/Cl, the two factors of clearance and volume of distribution can affect half-life. The more volume of distribution and the less clearance results in a longer half-life of a drug. Comparing volume of distribution and clearance in two genders imply no significant statistical difference between two genders. Yet numerically, volume of distribution and clearance of the drug is higher in women than in men. Changes of Vd can’t justify shorter half-life in women. On the other hand, comparison of clearance in two genders (2.17 L/kg/hr against 1.77 L/kg/hr) signifies that the speed of drug metabolism is more in women than in men and can justify a shorter half-life in women. In other words, in women the increase of clearance is more than the increase of Vd. Eventually, the half-life of the drug has been decreased to some extent. The area under the curve or the bioavailability after oral administration had been inversely associated with intrinsic clearance of liver. The Changes in intrinsic clearance leads to changes in the amount of the drug withdrawal in the first-pass effect in the liver, but has little impact on clearance and half-life of the drug.^27^ Therefore due to the lack of significant difference in the area under the curve, the liver metabolism capacity determining the intrinsic clearance of propranolol was not significantly different in two genders. As expected, systolic blood pressure of volunteers has decreased over time and after receiving propranolol. This decrease was significant in both genders unlike systolic blood pressure, changes in diastolic blood pressure over time was not significant. In this study, there were inclinations to an decrease in heart rate after single dose of the drug; similar results were obtained in study of Pharmacodynamic and pharmacokinetic of a single dose of 40 mg sublingual and oral propranolol in patients with high blood pressure.^28^

Comparing two genders no difference was observed in Pharmacodynamic parameters including heart rate, systolic and diastolic blood pressure. These results are compatible with pharmacokinetic parameters (volume of distribution, clearance and half-life) that were not significantly different between two genders.

In our study, the average clearance was 126.59 ml/h and the maximum and minimum clearance was 232.11 and 65.63, respectively. Several interpersonal changes are expected due to the effects of gender, race and age on the stable plasma concentration of the drugs metabolized by liver such as propranolol.^27,29^ In some studies it has been shown that oral clearance of propranolol is higher in African-Americans rather than the white people.^21,30,31^ In the study of Sowinski et al, the clearance in black and white people were 5036±4175 ml/min and 2854±879 ml/min respectively.^21^ Similar results were obtained by L-Isomer of propranolol.^30^ Unlike the study of Sowinski et, the average oral clearance was the same in black and white people in a study conducted in the United States (clearance range between 42.1 ml/min/kg and 54.5 ml/min/kg).^31^ The average clearance in our study (1.97 L/hr/kg equal to 32.8 ml/min/kg) was less than the mentioned studies. In Wilson and colleagues study clearance of propranolol was 1040±120 ml/kg/hr (17.3 ml/min/kg) that is in the range of the average clearance mentioned in references 0.96 L/kg/hr (equal to 16 ml/min/kg).^32^ Clearance in our study was more than the average clearance mentioned in the references (Table 4). The average volume of distribution had been 334.12 Lit (0.11±2.3 L/kg). Maximum and minimum volume of distribution was 633.73 Lit and 98.93 Lit, respectively. The volume of distribution obtained from our study had no significant difference with the average mentioned in the references. In the study conducted in Poland, the volume of distribution of propranolol was 5±1.2 L/kg, which was within the range of volume of distribution obtained in our study.^33^ In another study conducted in Malaysia by Zain-Hamid R and colleagues, volume of distribution following a single 20 mg dose of propranolol was 543.89±292.91 L which was more than the results from our study.^34^ Although in our study pharmacokinetic parameters of propranolol were examined as a single dose, but it has shown that the value of distribution with repeated doses decreases due to saturated tissue attachment of the drug.^34^ Half-life of propranolol in our study was 1.98±0.8 hour, which was less than the average mentioned in the references.^17,18^ In the study Castleden and George in England, half-life of the drug following the administration of a single oral dose of 40 mg in healthy volunteers was reported 217±13 minutes (equal to 3.61±0.22 hours).^35^ In another study in the same country, half-life of the drug was reported 4.7±0.9.^36^ Although there is a limitation in terms of the low number of participants in our study, the increased clearance and volume of distribution of subjects in our study suggest that the decrease of half-life was a consequence of a more increase in clearance rather than an increase in volume of distribution. In poor metabolizer people, following the administration of certain doses of the drug, there was more increase in the concentration of the drug, longer elimination half-life and an increase in the inhibition of beta. Therefore, administration of standard doses of beta blocker might cause the outbreak of dose-dependent side effects.^37^ One of propranolol metabolites is 4-Hydroxypropranolol resulted from drug oxidation in the route of P450 cytochrome. This metabolite in people with low metabolic capacity is less than that in extensive metabolizer people. Yet, in the study of Lennard and colleagues, it was demonstrated that the average plasma concentrations were similar after a single oral dose of 80 mg in two groups of people with low and normal metabolic capacity. This indicates that oxidation phenotype is not the main determinant of drug levels and dose-dependent side effect s.^38^ It seems that pharmacokinetic differences in our patients can’t justify their being over- responsive to beta blockers. Unexpectedly clearance of the drug studied volunteers was higher and their half-life was lower than the average mentioned in the references. Regarding the identifying of polymorphism in beta blockers,^39^ the differences in beta receptor expression in our population might justify pharmacodynamics differences. Several studies have been conducted on the polymorphism of beta- adrenergic receptors and its effects on changes in responding to the beta blocker treatment in different population. Also in the study of Parvez et al., it is indicated that people suffering from atrial fibrillation with common polymorphism of adrenergic beta-1 receptors, as Arg389Gly, give a better response to the treatments and need different doses of beta blocker and calcium blocker drugs.^40^ As long as hemodynamic responses to propranolol is different in various races and countries due to genetic differences, there has been conducted several studies in various populations. As an example it has been observed that people with genotype homozygote C/C188 take more benefits from treatment of liver cirrhosis with propranolol rather than patients with C/T188 genotype heterozygote.^41^ In another study it was observed that patients with cirrhosis and portal vein hypertension had different responses to propranolol, calculated by reduction of variceal pressure. That is, patients with Gly16-Glu/Gln27 genotype homozygote had shown more decrease in variceal pressure rather than Arg16-Gln27 homozygote people. Also, patients with heterozygote genotype had an intermediate response between within two homozygote genotypes.^42^ There are as well such studies about other beta blocker drugs. For instance, it is observed that in people with ADRB1 Arg389Gly polymorphism, there is a dose-response relation between plasma Renin activity and Metoprolol concentrations in healthy male Caucasian people. It seems that in these people (Gly/Gly), plasma Renin activity is inhibited in lower Metoprolol concentrations and they need lower doses to inhibit neurohormonal hyperactivity.^43^ In another study, it was observed that Arg 389Gly polymorphism has an important effect on the decrease in heart rate caused by carvedilol in patients with heart failure along with atrial fibrillation, but such effect is not true about Bisoprolol.^44^ Results have shown that homozygote mutant genotype ADRB1 1165G>C is associated with the increase in effectiveness of Metoprolol in treating hypertension, that suggest the selection of anti-hypertension according to genotype of patients.^45^ Furthermore, in Korean patients suffering from heart failure, it was observed that people with ADRB1 Gly389X genotype, have shown a better response to Bisoprolol compared to Arg389Arg genotype. These results show that treatment with beta- blocker goes to treatment of each person separately, on the basis of genotype.^46^ Another study indicates a 10 times difference in the effects of Esmolol on exercise- induced tachycardia in people with ADRB1 389 genotype.^47^ Furthermore, according to previous studies, in people with ADRb1-389 Arg/Arg genotype, a higher dose of beta blocker is needed to achieve a treatment response rather than in people with Gly genotype carriers.^48^

According to the results of the above-mentioned studies, the Pharmacodynamic difference of Propranolol in the present study may be associated with polymorphism of beta-adrenergic receptors in the population under study and its effect on the increase in responsiveness to this drug. It is worth to conduct future studies about polymorphism of beta receptors in order to investigate Pharmacodynamic differences in our population.

## Conclusion

According to the results of this study, it seems that pharmacokinetic differences are not able to explain over-responsiveness of our patients to propranolol. Pharmacodynamic differences in responding to beta blocker drugs by Renin secretion or having a different sensibility to beta receptors might play a role in making our population have a different response to propranolol.

## Acknowledgments

This study was an approved research project sponsored by Deputy of Research and Technology, Mazandaran University of Medical Sciences, to which the authors express their appreciation and thanks. We also declare our acknowledgment to the personnel of Fatemeh Zahra Hospital Laboratory as well as to all of the volunteers participated in this study.

## Ethical Issues

The approval number of the study was 2.3.84-458. The ethical standards were met according to Fifth revision of Declaration of Helsinki.

## Conflict of Interest

The authors report no conflicts of interest.
